# Improved diagnostic value by combining plasma PON1 level with tumor biomarkers in Colorectal Cancer patients

**DOI:** 10.7150/jca.45204

**Published:** 2020-09-17

**Authors:** Jingdan Zhang, Xiangling Yang, Lili Wei, Huiliu Tan, Junxiong Chen, Weiqian Li, Kawo Chan, Yixi Su, Lu Zhao, Suhua Hu, Shuoxian Zhong, Yanhong Xiao, Huanliang Liu

**Affiliations:** 1 Department of Clinical Laboratory, The Sixth Affiliated Hospital, Sun Yat-sen University, Guangzhou, Guangdong 510655, China.; 2 Guangdong Provincial Key Laboratory of Colorectal and Pelvic Floor Diseases, Guangdong Institute of Gastroenterology, The Sixth Affiliated Hospital, Sun Yat-sen University, Guangzhou, Guangdong 510655, China.

**Keywords:** Paraoxonase 1, biomarker, colorectal cancer, diagnostic value

## Abstract

The incidence of colorectal cancer (CRC) ranks third among all cancers in China and improvements in screening for CRC have an important impact on prevention and control of the disease. Paraoxonase 1 (PON1) is a calcium ion-dependent hydrolase that is widely distributed in tissue. Its diagnostic value in colorectal cancer has been reported, but the diagnostic value of combining PON1 with carcinoembryonic antigen (CEA), carbohydrate antigen 19-9 (CA19-9), carbohydrate antigen 12-5 (CA12-5) in colorectal cancer has not been evaluated. Experiments were carried out in a total of 284 CRC patients and 90 healthy controls. The primary cohort was randomly divided into training and validation sets. The levels of PON1 in plasma of CRC patients were significantly lower than that in the healthy controls (*P* < 0.001). It showed excellent diagnostic value with the AUC reaching 0.750 for the training set and 0.742 for the validation set. Furthermore, combining PON1 with CEA, CA12-5, CA19-9 could better classify CRC patients (AUC rising from 0.821, 0.716, 0.712 to 0.875, 0.817 and 0.814, respectively, in the training set, from 0.818, 0.581, 0.593 to 0.854, 0.770, and 0.772 in the validation set). In conclusion, PON1 can serve as a diagnostic biomarker for CRC and raise the sensitivity and specificity when incorporated with traditional tumor biomarkers.

## Introduction

Colorectal cancer (CRC) is one of the most prevalent cancers. The incidence of CRC ranks third among all cancers and the incidence is still rising 1-3. Most CRC patients are in advanced stages at the time of diagnosis. Studies have shown that screening individuals with risk factors can diagnose CRC at an early stage, reduce mortality, and even reduce the incidence by detecting and removing adenomas. The United States has begun to promote early screening for CRC since the 1980s. At present, the 5-year survival rate of CRC patients in the United States has reached 90%. This shows that screening for colorectal cancer has an important impact on morbidity and mortality 4.

In China, the two-step approach is commonly used in screening for CRC. The first step in screening is to identify the patient's level of risk for CRC, while the second step is to take a diagnostic screening for high-risk patients. Fecal occult blood test and high-risk questionnaire of medical history are used as screening methods at present, however their sensitivity and specificity are low 5,6. Colonoscopy remains the golden standard to diagnose CRC whose advantage is accurate and accessible to biopsy for pathological examination. Blood-based test is a new option. For example, although SEPT9 DNA in plasma has become a marker for CRC 7,8, its penetration still at a low rate due to its high price. Therefore, there is a need for methods with high sensitivity, high specificity and patient compliance.

Paraoxonase 1 (PON1) is an aromatic esterase that catalyzes the hydrolysis of phosphate linkages. It is mainly synthesized by the liver and released into the blood. High density lipoprotein (HDL) is used as a carrier in the blood, which works by tightly binding to ApoAI 9. Some of altered lipid molecules may be potential biomarkers of CRC risk, development and progression 10. This enzyme inhibits low density lipoprotein (LDL) peroxidation and decomposes LDL peroxidation products into non-toxic small molecules, thereby attenuating oxidative stress in macrophages 11,12. The levels of PON1 in plasma may be related to CRC 13. LDL peroxidation product increases when PON1 is of low level, resulting in an excess of reactive oxygen species, causing imbalance between antioxidant system and oxidant system. Studies have reported the significance of PON1 in diagnosing CRC in European populations, and revealed the relationship between PON1 gene polymorphism and PON1 enzyme activity 14,15. However, research on the significance of PON1 and the combination of PON1 and commonly used biomarkers CEA, CA12-5, CA19-9 to diagnose CRC in Chinese population is still unclear. Therefore, this study focused on the plasma PON1 levels in Chinese population and the value of PON1 combined with tumor biomarkers in the diagnosis of colorectal cancer.

## Materials and methods

### Sample collection

Blood samples from 284 CRC patients and 90 healthy controls were collected from the Sixth Affiliated Hospital, Sun Yat-sen University between September 2018 and June 2019. The study was approved by the Ethics Committee of the Sixth Affiliated Hospital, Sun Yat-sen University (ethical approval number 2018ZSLYEC-008). The research was done according to the Declaration of Helsinki. All patients were included according to the medical and pathology reports. Patients with missing data and who accepted any chemotherapy or radiotherapy were excluded in our study. Healthy controls were selected based on their age and gender to match CRC patients to the greatest extent. Plasma samples in EDTA tubes were collected within 6 h from drawn blood. Blood samples were centrifuged at 3,000 r/min for 2 min, then transfer the supernatants into 1.7 mL EP tubes and immediately stored at -80 ºC. Plasma samples were centrifuged at 130,000 r/min for 10 min at 4 ºC before experiment.

## Methods

Clinical and pathological data were investigated. Serum tumor biomarkers were measured by chemiluminescent enzyme immunoassay (CLEIA) on an Architect (Abbott Laboratories, Chicago, IL, USA). PON1 was measured by enzyme-linked immunosorbent assay (ELISA) using reagent kits (RayBiotech, Guangzhou, Guangdong, China). ELISA was performed according to the manufacture's instruction.

### Statistical analysis

All statistical analyses were performed using the SPSS 26.0, MedCalc 19.0 and GraphPad Prism 8.0. The comparisons of CEA, CA12-5, CA19-9, and PON1 between the Training set and the Validation set were assessed using Mann-Whitney U test. Receiver operating characteristic (ROC) curves were plotted and the areas under the curve (AUC) were calculated. The efficacy of PON1 for diagnosis was evaluated by AUC. Cut-off value was defined as the value with the maximization of the Youden index. Furthermore, sensitivity (Sen), specificity (Spe), positive predictive value (PPV), and negative predictive value (NPV) were used to compare the diagnostic value among PON1, CEA, CA19-9, CA12-5. Logistic regression model was binomial fitted to combine diagnostic performance of biomarkers. All statistical tests were two-sided and *P* < 0.05 was considered statistically significant.

## Results

### Characteristics of the subjects

We have collected several clinicopathologic features of the 284 CRC patients, including age, gender, TNM stage and tumor location (**Table 1**). According to P values, the levels of PON1 were not obviously correlated with age, T classification, N classification, metastasis, clinical stage and tumor location in CRC patients. There could be a significant association between gender and the levels of PON1 (*P* = 0.014), which was lower in males than in females. Baseline characteristics were compared in training and validation sets (**Table 2 & Table 3**). All *P* values > 0.05 indicated that grouping was valid and able to be analyzed in the next step.

### Correlation of PON1 and CRC according to plasma levels

To assess the relationship between plasma PON1 levels and CRC, we calculated Spearman's correlation coefficient. For PON1, the correlation coefficient constant *r* was -0.510 (*P* < 0.001). For CEA, CA12-5 and CA19-9, the correlation coefficient constant *r's* were 0.364 (*P* < 0.001), 0.182 (*P* = 0.001) and 0.198 (*P* < 0.001). These data suggested that CEA and PON1 are most likely to diagnose CRC.

### Decreased plasma PON1 levels in colorectal cancer patients

**Figure 1:**
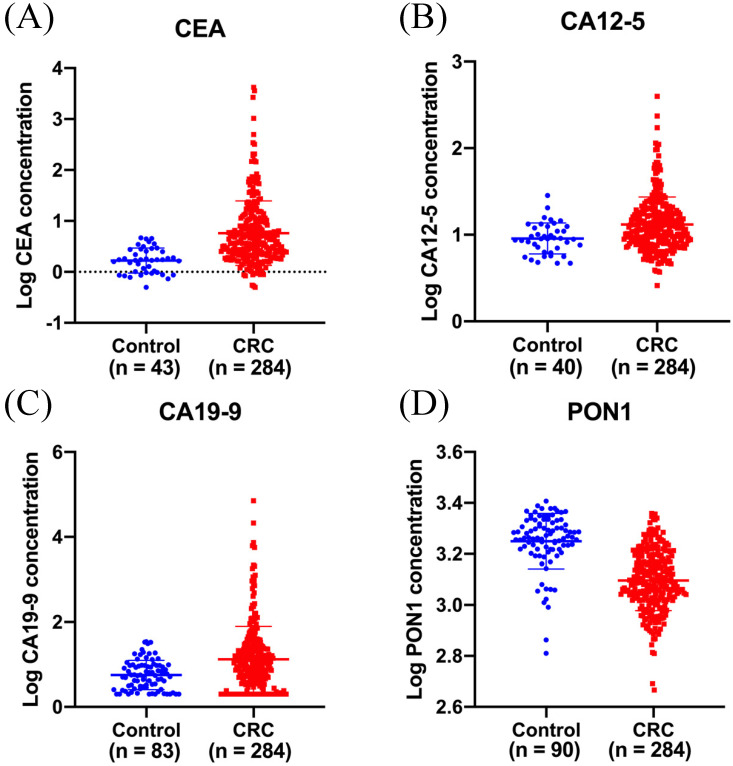
** Plasma PON1 levels are decreased in colorectal cancer patients.** CEA **(A)** CA12-5 **(B)**, CA19-9 **(C)** levels in serum were measured in 284 cancer patients and part of healthy controls. **(D)**The plasma PON1 levels were measured in 284 cancer patients and 90 healthy controls.

ELISA was performed to evaluate plasma PON1 levels in 284 CRC patients and 90 healthy controls. The median plasma concentration of PON1 was 1,226 (1,049-1,528) ng/mL in CRC patients and 1,840 (1,651-2,111) ng/mL in control subjects (**Figure 1D**). In order to reduce the variation among all the values, we have transformed the raw data by logarithmic function. The median serum concentration of CEA was 3.9 (2.2-10.3) ng/mL in CRC patients and 1.7 (1.0-2.8) ng/mL in control subjects (**Figure 1A**). The median serum concentration of CA12-5 was 11.6 (8.0-18.0) U/mL in CRC patients and 9.0 (6.5-12.4) U/mL in control subjects (**Figure 1B**). The median serum concentration of CA19-9 was 10.3 (3.6-26.2) U/mL in CRC patients and 5.5 (2.7-9.5) U/mL in control subjects (**Figure 1C**).

### Diagnostic value of individual PON1, CEA, CA12-5 and CA19-9 levels for CRC

**Figure 2:**
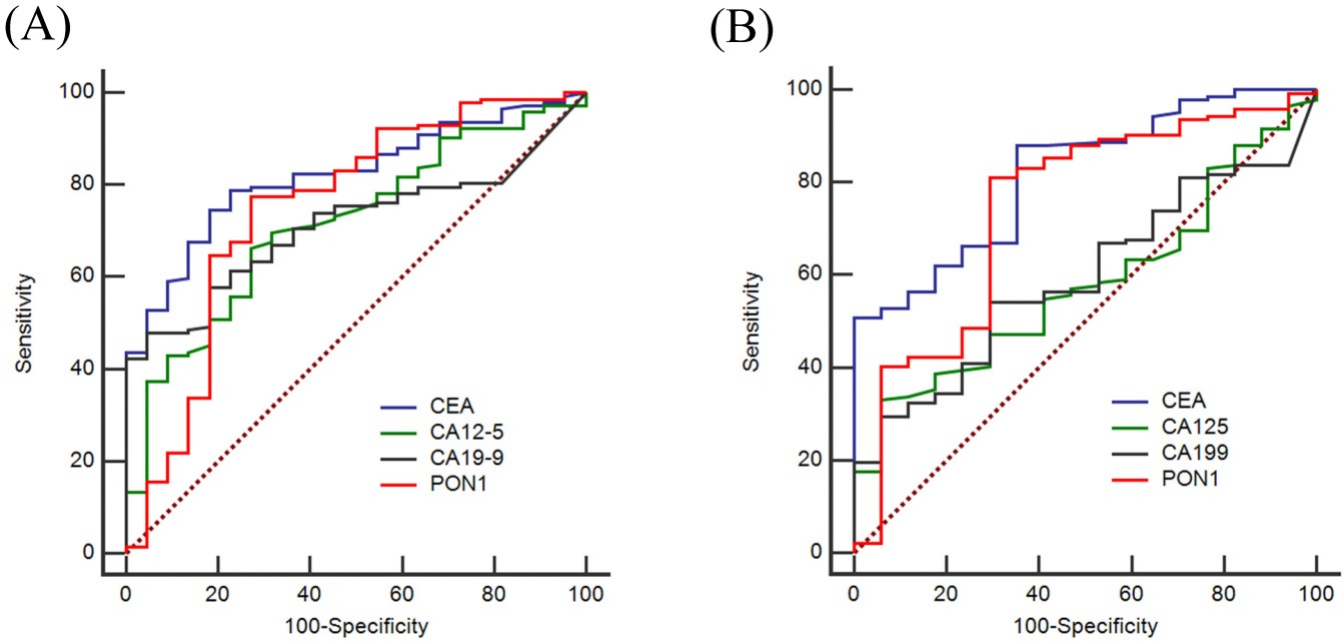
** ROC curves of using CEA, CA12-5, CA19-9 and PON1 alone in training and validation sets.** To certify the utility of PON1 in the diagnosis of colorectal cancer, we plotted ROC curves and determine cut-off values. **(A)** Diagnostic outcomes for PON1, CEA, CA12-5 and CA19-9 alone in Training set. **(B)** Diagnostic outcomes for PON1, CEA, CA12-5 and CA19-9 alone for the validation set.

To evaluate the diagnostic value of PON1 levels, we plotted ROC curves and calculated the sensitivity and specificity of PON1, CEA, CA12-5 and CA19-9. Based on the training set as shown in **Figure 2A**, the AUCs of PON1 and CEA reached 0.750 (95% CI: 0.624-0.876), and 0.821 (95% CI: 0.745-0.896), respectively, whereas the AUCs for CA12-5 and CA19-9 were only 0.716 (95% CI: 0.612-0.820) and 0.712 (95% CI: 0.627-0.796). **Table 4** demonstrated the sensitivity and specificity of PON1 was 0.775 and 0.727 based on the optimal cut-off (1,556). The optimal cut-offs of CEA, CA12-5, CA19-9 were 2.2, 9.7 and 10.0. In the validation set (**Figure 2B**), the AUCs of CEA, CA12-5, CA19-9, PON1 were 0.818 (95% CI: 0.725-0.910), 0.581 (95% CI: 0.462-0.700), 0.593 (95% CI: 0.476-0.711) and 0.742 (95% CI: 0.607-0.877) which could lead to the same conclusion as the training set. The diagnostic value of the validation set is shown in **Table 5.**

### Diagnostic value of PON1, CEA, CA12-5, CA19-9 levels combination for CRC

**Figure 3:**
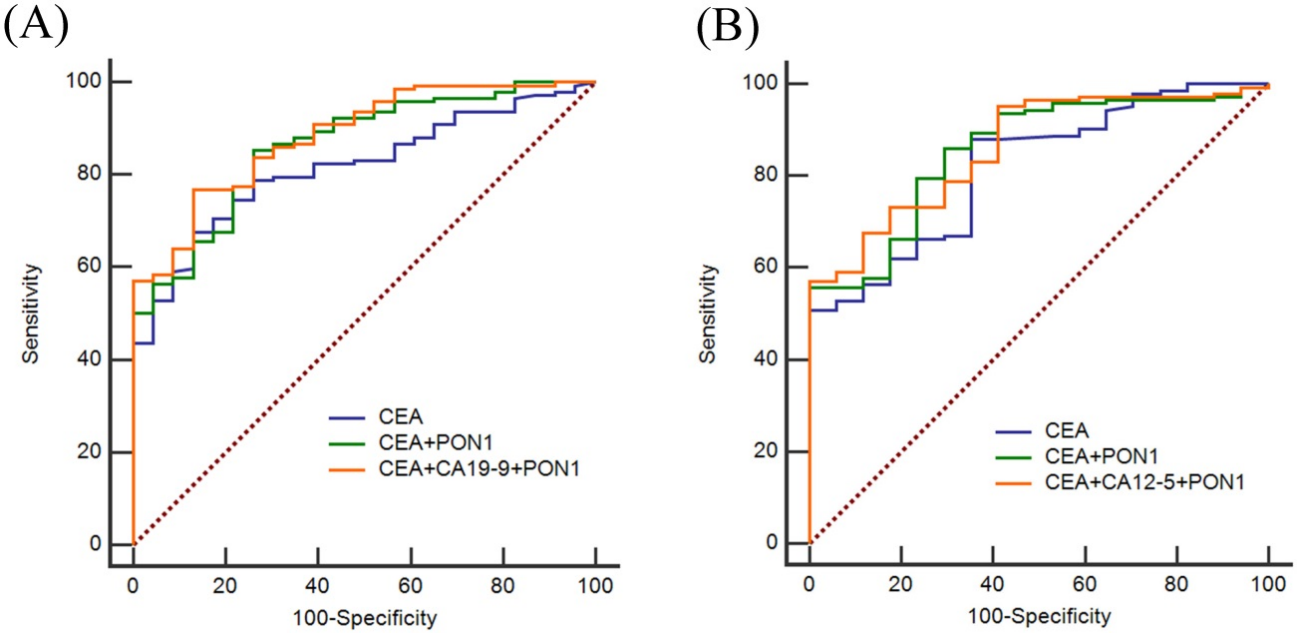
** ROC curves of using CEA, CA12-5, CA19-9 and PON1 in combination in training and validation sets.** (**A**) Best diagnostic outcomes for PON1, CEA, CA12-5 and CA19-9 in combination in Training set. (**B**) Best diagnostic outcomes for PON1, CEA, CA12-5 and CA19-9 in combination in Validation set.

CEA, CA12-5 and CA19-9 are the most commonly used clinical index to screen cancer, so we also assessed the diagnostic value of CEA, CA12-5, CA19-9 in combination with PON1 in CRC. As shown in **Figure 3**, by combining with PON1, the overall AUCs increased. In the training set, the AUC was 0.821 for CEA and 0.875 for combination of PON1 and CEA. The specificity reached 0.852 and NPV reached 0.611 which is higher than combining CEA with CA12-5 or CA19-9. The results in the validation set confirm the performance of PON1 as a plasma biomarker to increase the diagnostic value of CRC detection, and suggest that the levels of CEA and PON1 combination could be better screened CRC than either CEA alone or combined with CA12-5 or CA19-9. The sensitivity and specificity of CEA reached 0.859 and 0.750 in the validation set when combined with PON1.

## Discussion

Liquid biopsy is one of the most promising screening methods because of its simplicity, convenience, and minimally invasiveness. It provides a faster route for diagnosis, monitoring and prognosis of colorectal cancer 16. At present, clinical practice mainly uses CEA and CA19-9 for CRC screening. The increment of CA12-5 is commonly observed in epithelial tumors. Due to their low sensitivity and specificity, there is an urgent need to explore new biomarker or validate most potential biomarkers.

Previous studies have confirmed that PON1 is a calcium ion-dependent hydrolase that is widely distributed in tissue. It mainly expresses in the liver and assembled with HDL. The significant association between gender and the levels of PON1 (*P* = 0.014) may related to the positive correlation between PON1 and HDL, and the difference in HDL between male and female 17. Recent studies have shown that low PON1 activity is associated with increased risk of breast cancer 18 and risk of gastric cancer metastasis 19. The antioxidant capacity of PON1 is related to its peroxidase activity 9. Low activity of PON1 will result in excess reactive oxygen species expose individuals to higher oxidative stress and increases risk of cancer 20,21. Changes in plasma PON1 levels are also associated with liver cell destruction and chronic inflammation 22. Its diagnostic value in endometrial cancer, colorectal cancer, etc. has also been reported 23,24. In this study, we found that PON1 also has diagnostic value for CRC in the Chinese population which is consistent with previous research.

When CEA, CA19-9, CA12-5 are combined with PON1, their AUCs are improved to various extents. The AUC of CEA improved from 0.821 to 0.875 in Training set and from 0.818 to 0.854 in Validation set. Improvement in specificity can reduce the rate of misdiagnosis without sacrificing sensitivity. These findings indicated that common biomarkers combined with PON1 could be better screened for CRC than used alone. We inferred the results may be related to obesity and lipid metabolism. Obesity demonstrates a major public health issue because of its derived risk factors which are strongly associated with many diseases including CRC 25. Studies have shown that decreased HDL is an important determinant of lipid peroxidation and it is of lower level in obese people than in others 26. Reverse cholesterol transport mechanism is defined as the removal of cholesterol from peripheral macrophage foam cells, via HDL, and cholesterol transportation to the liver for excretion 27. Oxidative modification of LDL is involved in the production of inflammatory cytokines which associated with the cancer development 28. PON1 inhibits LDL peroxidation and decomposes LDL peroxidation products into non-toxic small molecules in which way related to cancer occurrence 11,12.

Here, the limitations of this study have to be mentioned. Firstly, all cases of colorectal cancer are from a hospital with well reputation for gastrointestinal diseases. The findings need further confirmation in multi-center though an internal validation was performed. Secondly, part of the tumor biomarker data was missing in the healthy control group. Finally, this study is a retrospective study that requires further design of a prospective study to demonstrate that PON1 combined with tumor biomarkers can improve the diagnostic efficacy of colorectal cancer.

## Figures and Tables

**Table 1:** Relationship between PON1 and the clinicopathological variables in 284 CRC patients

Variable	n	PON1 (ng/mL), median (P_25_-P_75_)	*P* value
**Gender**			
Male	180	1,181 (1,020-1,471)	0.014
Female	104	1,273 (1,109-1,607)	
**Age (y)**			
<60	134	1,217 (1,071-1,485)	0.747
≥60	150	1,239 (1,021-1,542)	
**T classification**			
T1+T2	52	1,279 (1,110-1,576)	0.126
T3+T4	232	1,205 (1,041-1,493)	
**N classification**			
No	116	1,245 (1,098-1,529)	0.182
Yes	168	1,211 (999-1,528)	
**Metastasis**			
No	229	1,227 (1,064-1,491)	0.465
Yes	55	1,214 (961-1,556)	
**Stage**			
I+II	103	1,219 (1,069-1,477)	0.718
III+IV	181	1,243 (1,039-1,544)	
**Location**			
Colon	135	1,200 (1,030-1,484)	0.078
Rectum	149	1,273 (1,068-1,561)	

**Table 2:** Baseline characteristics of CRC patients in both training and validation sets

Variable	Training set (n = 142)	Validation set (n = 142)	*P* value
Age (y)	61 (28-86)	60 (22-88)	0.478
**Gender**			
Male/Female	93/49	87/55	0.539
**T classification**			
T1/T2/T3/T4	7/17/79/39	9/19/88/26	0.328
CEA (ng/mL)	3.6 (0.5-4,185.8)	4.6 (0.9-3,602.6)	0.333
CA12-5 (U/mL)	13.5 (2.6-398.1)	10.5 (3.8-235.5)	0.061
CA19-9 (U/mL)	8.4 (2.0-70,923.8)	10.8 (2.0-5,674.6)	0.446
PON1 (ng/mL)	1,237 (463-2,285)	1,222 (645-2,258)	0.470

**Table 3:** Baseline characteristics of healthy controls in both training and validation sets

Variable	Training set (n = 45)	Validation set (n = 45)	*P* value
Age (y)	55 (27-78)	58 (29-80)	0.234
Gender (Male/Female)	24/21	24/21	1.000
CEA (ng/mL)	1.7 (0.5-4.5)	1.7 (0.7-4.7)	0.488
CA12-5 (U/mL)	8.7 (4.7-28.5)	9.7 (4.7-20.5)	0.411
CA19-9 (U/mL)	5.8 (2.0-33.4)	4.9 (2.0-34.6)	0.931
PON1 (ng/mL)	1,869 (646-2,552)	1,820 (729-2,388)	0.818

**Table 4:** The diagnostic value of CEA, CA12-5, CA19-9 and PON1 in the training set

	Sensitivity	Specificity	PPV	NPV	AUC	*P* value
CEA	0.746	0.828	0.930	0.507	0.821	< 0.001
CA12-5	0.662	0.727	0.888	0.407	0.716	0.001
CA19-9	0.479	0.955	0.986	0.373	0.712	0.001
PON1	0.775	0.727	0.902	0.508	0.750	< 0.001

**Table 5:** The diagnostic value of CEA, CA12-5, CA19-9 and PON1 in the validation set

	Sensitivity	Specificity	PPV	NPV	AUC	*P* value
CEA	0.782	0.600	0.860	0.466	0.818	< 0.001
CA12-5	0.585	0.500	0.790	0.280	0.581	0.275
CA19-9	0.535	0.780	0.884	0.347	0.593	0.209
PON1	0.911	0.423	0.832	0.594	0.742	0.001

**Table 6:** The diagnosis value of CEA, CA12-5, CA19-9 and PON1 in combination to diagnose CRC in training set

Combination	Sensitivity	Specificity	PPV	NPV	AUC	*P* value
CA12-5+CA19-9	0.606	0.955	0.977	0.434	0.796	< 0.001
CEA+CA19-9	0.648	0.957	0.979	0.462	0.851	< 0.001
CEA+CA12-5	0.796	0.864	0.950	0.574	0.867	< 0.001
PON1+CEA	0.852	0.739	0.910	0.611	0.875	< 0.001
PON1+CA12-5	0.894	0.682	0.901	0.674	0.817	< 0.001
PON1+CA19-9	0.838	0.762	0.915	0.596	0.814	< 0.001
PON1+CA12-5+CA19-9	0.761	0.818	0.931	0.521	0.864	< 0.001
PON1+CEA+CA19-9	0.768	0.870	0.948	0.542	0.898	< 0.001
PON1+CEA+CA12-5	0.894	0.773	0.927	0.700	0.893	< 0.001
CEA+CA12-5+CA19-9	0.824	0.864	0.951	0.609	0.884	< 0.001
PON1+CEA+CA12-5 +CA19-9	0.817	0.864	0.951	0.600	0.909	< 0.001

**Table 7:** The diagnosis value of CEA, CA12-5, CA19-9 and PON1 in combination to diagnose CRC in validation set

Combination	Sensitivity	Specificity	PPV	NPV	AUC	*P* value
CA12-5+CA19-9	0.648	0.722	0.876	0.390	0.669	0.023
CEA+CA19-9	0.542	1.000	1.000	0.413	0.829	< 0.001
CEA+CA12-5	0.894	0.647	0.888	0.659	0.831	< 0.001
PON1+CEA	0.859	0.750	0.917	0.630	0.854	< 0.001
PON1+CA12-5	0.838	0.722	0.902	0.582	0.770	< 0.001
PON1+CA19-9	0.852	0.854	0.945	0.644	0.772	< 0.001
PON1+CA12-5+CA19-9	0.894	0.722	0.907	0.681	0.792	< 0.001
PON1+CEA+CA19-9	0.838	0.722	0.902	0.582	0.860	< 0.001
PON1+CEA+CA12-5	0.570	1.000	1.000	0.425	0.861	< 0.001
CEA+CA12-5+CA19-9	0.845	0.706	0.902	0.593	0.840	< 0.001
PON1+CEA+CA12-5 +CA19-9	0.761	0.824	0.931	0.521	0.867	< 0.001
